# Epidemiology of enterotoxigenic *Escherichia coli* and impact on the growth of children in the first two years of life in Lima, Peru

**DOI:** 10.3389/fpubh.2024.1332319

**Published:** 2024-03-22

**Authors:** Monica J. Pajuelo, Sassan Noazin, Lilia Cabrera, Angie Toledo, Mirza Velagic, Lucero Arias, Mayra Ochoa, Lawrence H. Moulton, Mayuko Saito, Robert H. Gilman, Subhra Chakraborty

**Affiliations:** ^1^Laboratorio Microbiología Molecular – Laboratorios de Investigación y Desarrollo, Facultad de Ciencias e Ingeniería, Universidad Peruana Cayetano Heredia, Lima, Peru; ^2^Department of International Health, Bloomberg School of Public Health, Johns Hopkins University, Baltimore, MD, United States; ^3^Asociación Benéfica Prisma, Lima, Peru; ^4^Department of Virology, Tohoku University Graduate School of Medicine, Sendai, Japan

**Keywords:** diarrhea, enterotoxigenic *E. coli*, disease burden, growth in children, Peru, birth cohort, epidemiology, colonization factors

## Abstract

**Background:**

Enterotoxigenic *E. coli* (ETEC) is a leading cause of diarrheal morbidity and mortality in children, although the data on disease burden, epidemiology, and impact on health at the community level are limited.

**Methods:**

In a longitudinal birth cohort study of 345 children followed until 24 months of age in Lima, Peru, we measured ETEC burden in diarrheal and non-diarrheal samples using quantitative PCR (LT, STh, and STp toxin genes), studied epidemiology and measured anthropometry in children.

**Results:**

About 70% of children suffered from one or more ETEC diarrhea episodes. Overall, the ETEC incidence rate (IR) was 73 per 100 child-years. ETEC infections began early after birth causing 10% (8.9–11.1) ETEC-attributable diarrheal burden at the population level (PAF) in neonates and most of the infections (58%) were attributed to ST-ETEC [PAF 7.9% (1.9–13.5)] and LT + ST-ETEC (29%) of which all the episodes were associated with diarrhea. ETEC infections increased with age, peaking at 17% PAF (4.6–27.7%; *p* = 0.026) at 21 to 24 months. ST-ETEC was the most prevalent type (IR 32.1) with frequent serial infections in a child. The common colonization factors in ETEC diarrhea cases were CFA/I, CS12, CS21, CS3, and CS6, while in asymptomatic ETEC cases were CS12, CS6 and CS21. Only few (5.7%) children had repeated infections with the same combination of ETEC toxin(s) and CFs, suggested genotype-specific immunity from each infection. For an average ETEC diarrhea episode of 5 days, reductions of 0.060 weight-for-length z-score (0.007 to 0.114; *p* = 0.027) and 0.061 weight-for-age z-score (0.015 to 0.108; *p* = 0.009) were noted in the following 30 days.

**Conclusion:**

This study showed that ETEC is a significant pathogen in Peruvian children who experience serial infections with multiple age-specific pathotypes, resulting in transitory growth impairment.

## Introduction

Enterotoxigenic *E. coli* (ETEC) causes significant global morbidity and mortality in low-and middle-income countries (1–3). Despite being an important pathogen, the paucity of data on ETEC disease burden, particularly at the national and sub-national levels, has created uncertainties in the reported ETEC-associated morbidity and mortality estimates (4, 5). This lack of data called for a widespread improvement in the quality and quantity of data, including improved surveillance systems, and using appropriate diagnostic tools to reveal the disease burden (6, 7). The region-specific estimates for the acute and long-term burden of ETEC would guide funders and public health officials to make evidence-based decisions to design effective vaccines and age-appropriate vaccination schedules for the regions with high pathogen burden (8).

ETEC toxin, heat-labile (LT) and colonization factors (CFs) are the primary target antigens in ETEC vaccine development (9–12). After adherence to the intestinal mucosa, ETEC produce one or both of two enterotoxins, heat-labile enterotoxin (LT) and heat-stable enterotoxin (ST). There are two types of ST, STa and STb. ETEC strains isolated from humans produce STa, and STb predominates in ETEC from animals. There are two subtypes of STa: STh and STp (1). STh (human) is a short (19-amino-acid) peptide and poorly immunogenic; thus, it itself cannot be used as a vaccine component (13, 14) and is not included in the most advanced ETEC vaccine candidates (9–12); although, there are current approaches to include STh in the vaccine candidates (15, 16). Evidence about the role of ETEC strains producing STp (porcine) as the only enterotoxin in causing moderate to severe diarrhea is contradictory (17, 18). While the prevalence of the ETEC toxin types, CFs and O serogroups varies substantially by region (19, 20), conflicting data are available on the relative importance of these virulence factors in protection from ETEC (1, 21). Data on the sequelae of the infections associated with types of ETEC by age are needed to elucidate the role of these virulence factors. Understanding the shedding duration of the pathotypes of ETEC in stool following infection could bring insight to our planning for a reduction in transmission of this enteric disease. The effect of ETEC diarrhea on immediate and long-term growth faltering is an important aspect that needs to be further studied (22, 23).

We conducted a prospective birth cohort study of Peruvian children in a censused population in Lima, Peru, to determine the natural history of ETEC infections, age specific ETEC disease burden, the impact of ETEC infections on morbidity and nutritional status during the first two years of life and identify the ETEC vaccine antigens and natural protection relevant to this region.

## Materials and methods

### Participants and procedures

A total of 345 newborns within 35 days and their mothers were enrolled in a staggered fashion to control for seasonality in a peri-urban community in Lima from 2016 to 2019. The children were followed until they were 24 months old. The children who presented with severe disease or a birth weight < 1,500 g were excluded.

The diarrheal episodes, and morbidity data were recorded by the fieldworkers through daily home visits. The diarrhea severity scoring criteria were based on the CODA index (24). CODA uses the number of days with fever, anorexia, vomiting, the number of liquid stools, and the maximum number of stools in a 24-h period during the episode. The anthropometry (length and weight) of the children was measured every month. A stool sample was collected every week and during the diarrhea episode and transported to the laboratory in insulated cooler box. All stools from diarrhea episodes and one routine stool every three months were tested for ETEC from DNA extracted from the stool followed by qPCR for the LT, STh, and STp genes (7, 25). Samples with a cycle threshold (Cq) value of <40 for LT, STh, or STp were considered positive for ETEC. We also calculated the incidence rates with a more stringent cutoff, Cq35, and presented the difference in the Supplementary Table S1. Conventional culture for *E. coli* was performed on MacConkey agar from the collected stool, followed by PCR for LT, STh, and STp genes of five selected *E. coli* colonies per sample (7). Thirteen colonization factors (CFA/I, CS1-8, CS12, CS21, CS17, CS17/19) were tested from the ETEC isolates or from DNA isolated from stool (when isolates were not available) using multiplex PCR (26) and confirmation with simplex PCR. To further characterize the ETEC strains, we detected the O serogroups. Randomly selected 44 ETEC isolates from ETEC diarrhea stool were tested using slide agglutination with poly and monoclonal O-antigen antisera (Denka Seiken, Japan) (27). Stools from randomly selected 147 ETEC positive cases (98 asymptomatic and 50 diarrheal) were tested for co-pathogens norovirus (GI and GII) (28), rotavirus (29), sapovirus (30), adenovirus (31), *Shigella* spp. (7), and *Campylobacter* spp. (32) using qPCR. For blood grouping, Hematest A1 (Diagast) kit was used. The secretory status was performed on saliva samples using a direct ELISA assay to identify the presence of H antigen. The sample was considered positive when the absorbance was at least 4 times greater than the absorbance of the negative control.

#### Statistical analysis

The cumulative incidence and 95% CI of ETEC diarrhea and infections were estimated using Kaplan Meier. A non-diarrhea ETEC episode was defined as a positive sample collected more than 30 days after a diarrhea episode ended and at least 15 days before a diarrhea episode started. The ETEC-attributable diarrheal burden at the population level (PAF) (33) over two years was estimated by age group at three-month intervals. PAF is defined as the fraction of all cases of a particular disease or other adverse condition in a population that is attributable to a specific exposure. ETEC shedding duration by toxin types was compared using the Kruskal Wallis test and *post hoc* Dunn’s test. Toxin and CFs-specific protection from repeated infections was estimated using time-dependent variable Cox proportional hazards regression for multiple event data using the age of children for the model timeline, accounting for intra-child correlation using standard robust variance estimates. Diarrhea and non-diarrhea samples were compared using GEE logistic regressions with exchangeable correlation to account for intra child correlation. We evaluated the association between the total number of ETEC episodes over the follow-up and anthropometric measurements at the end of the follow up by means of linear regression models. The velocity model was used to assess the more immediate association between the proportion of ETEC diarrhea days during any anthropometric measurement interval and the average change in anthropometric measurements over the following measurement interval. Study definitions and analysis details are provided in the Supplementary material.

## Results

### The burden of ETEC infection and diarrhea

Out of 345 enrolled children, 259 (75.1%) completed follow-up until 24 months of age, with a total follow-up period of 201,520 days (Table 1). ETEC was detected in 393 (25.6%) of the diarrhea episodes tested. The average number of ETEC diarrheal days was 5.3 days/episode. The number of ETEC diarrhea episodes was not different between girls and boys, accounting for intra-child correlation and follow-up time (*p* = 0.941). Most ETEC episodes were moderate to severe diarrhea (86.7%); 6.4% were persistent diarrhea, of which 44% episodes lasted for ≥14 days (maximum 26 days); ~21% of cases were with fever and vomiting each. No significant differences were noted between the clinical outcomes of the ETEC positive and negative diarrhea episodes (Table 2). Among the routine non-diarrheal specimens, ETEC was detected in 373 (22.1%) [highest 30% at 21–24 months and lowest 7% at 0–3 months]; 18.5% in the first and 30% in the second year of life.

**Table 1:** Descriptive statistics of the cohort.

Total children enrolled	Male to female ratio	At least 50% of days had only breast feeding in <6 months	Children completed 24 months of follow up	Routine surveillance stool		Diarrheal Episodes	
				Stools tested/collected (Tested every 3 month)	ETEC positive /stool tested	Diarrheal stools tested/episodes (%)	ETEC positive/stool tested
345	0.94	251/345 (72.8%)	259 (75.1%)	1686/28,809 (5.9%)	373/1,686 (22.1%)	1,536/1,749 (87.8%)	393/1,536 (25.6%)

**Table 2:** Clinical outcomes of the ETEC positive and ETEC negative diarrhea episodes.

Diarrhea	ETEC positive *n* = 393	ETEC negative *n* = 1,143	*p* value
Acute (<7 days)	368 (93.6%)	1,062 (92.9%)	0.625
Persistent (≥7 days)	25 (6.4%)	81 (7.1%)	
Mild (CODA index 0)	70 (17.8%)	216 (18.9%)	0.627
Moderate (CODA index 1–6)	292 (74.3%)	852 (74.5%)	
Severe (CODA index ≥7)	31 (7.9%)	75 (6.6%)	
Fever (yes/no)	80 (20.4%)	242 (21.2%)	0.732
Vomiting (≥1/24 h)	85 (21.6%)	242 (21.2%)	0.849

Of the children with blood group and secretor status detected, the majority were blood type O [80% (221 of 277)] and secretors [98.5% (329 of 334)] with no significant association with the number or severity of ETEC diarrhea (see Supplementary Figure S1 and Supplementary Table S2).

**Figure 1:**
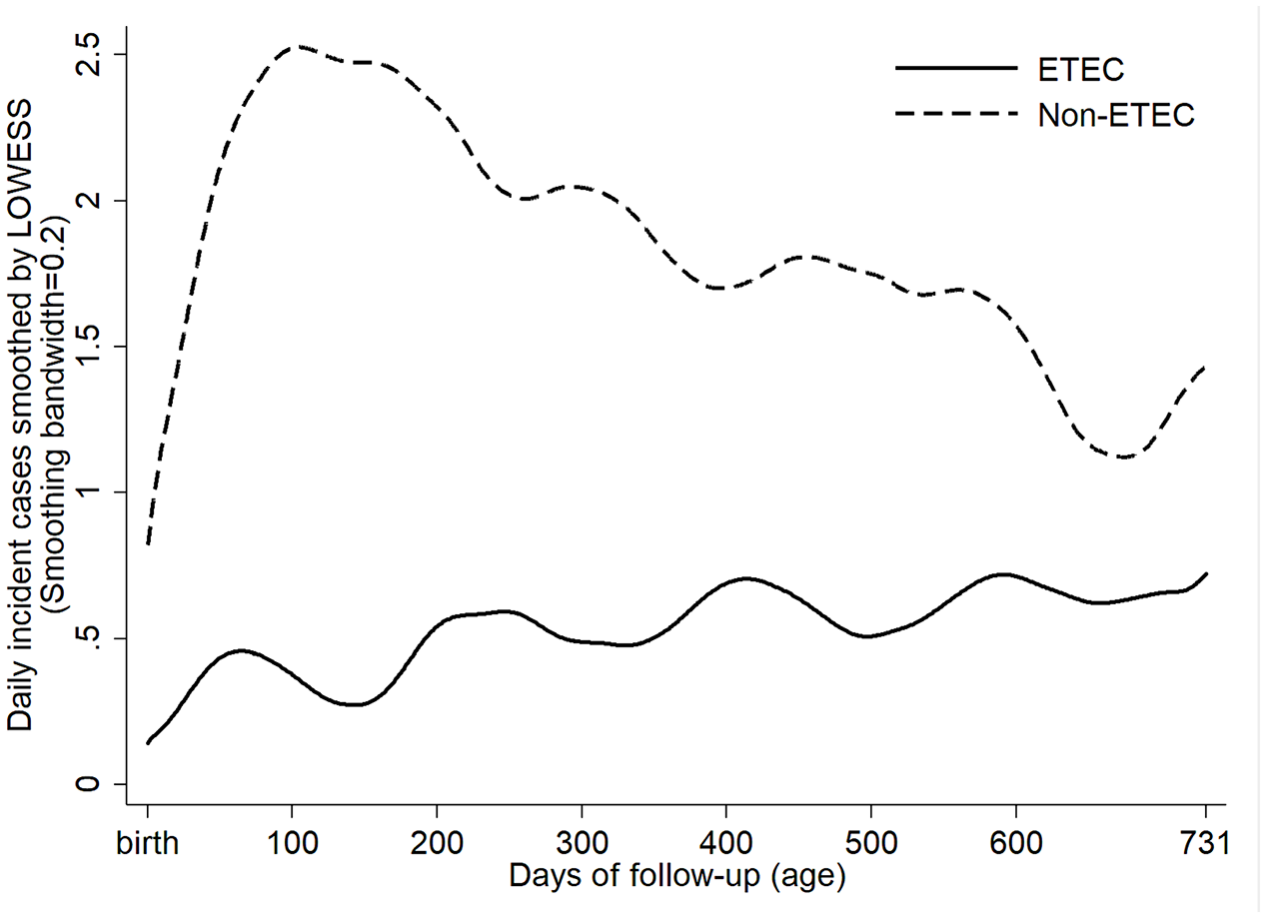
Daily incident cases of ETEC positive and ETEC negative diarrhea episodes. Daily incident cases of ETEC positive and non-ETEC diarrhea from birth to the end of the follow-up, smoothed by LOWESS (locally weighted scatterplot smoothing).

**Figure 2:**
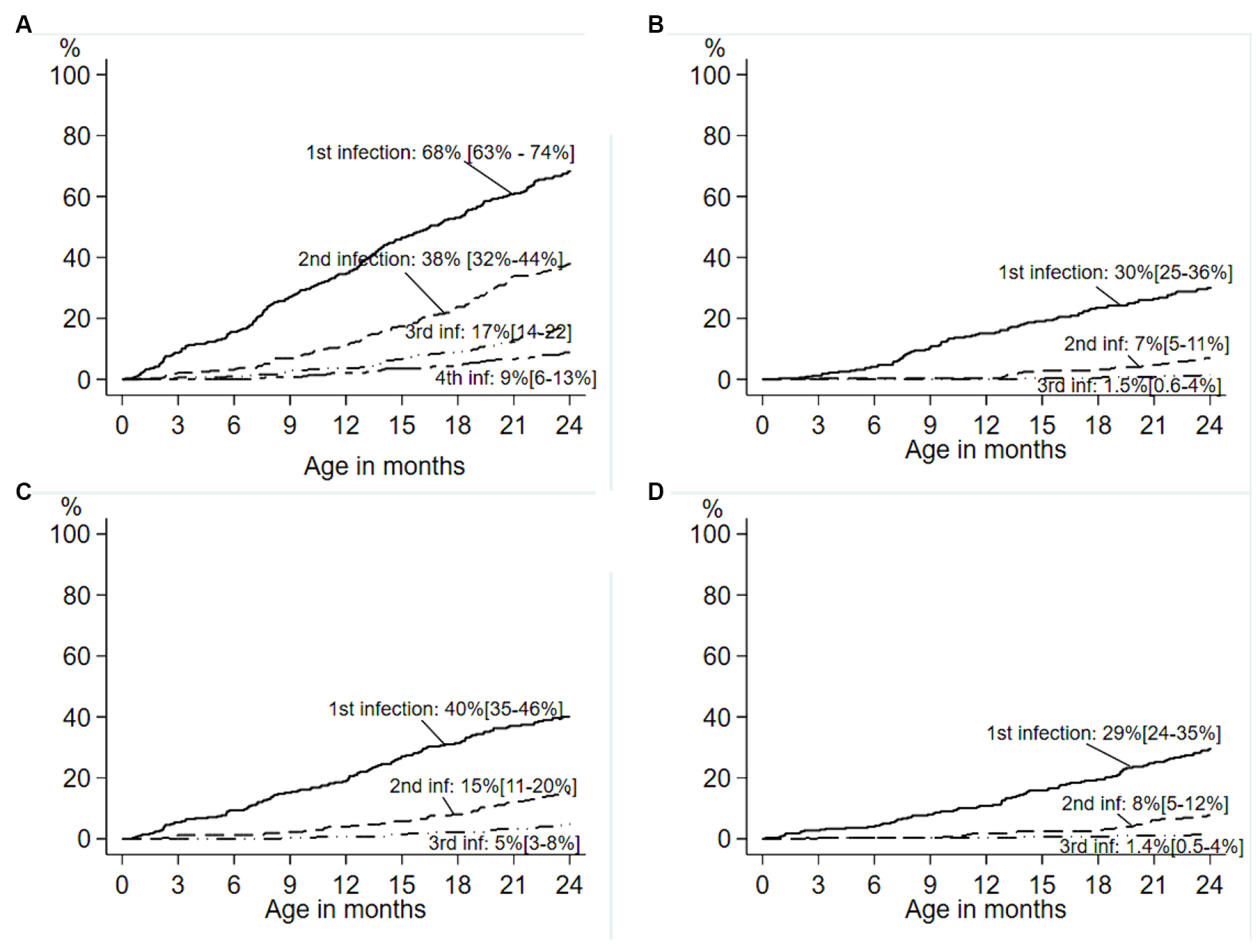
Cumulative incidence of first and subsequent ETEC diarrhea in the birth cohort. Cumulative incidence of first and subsequent ETEC diarrhea episodes in the birth cohort of 345 children. **(A)** Survival curves showing cumulative incidence of the first through fourth ETEC diarrhea episodes during the first two years of life. **(B–D)** Toxin-specific survival curves showing the cumulative incidence of the first through third episodes of ETEC diarrhea in the children 0–2 years age. **(B)** LT-ETEC; **(C)** ST-ETEC; **(D)** LT + ST-ETEC. Percentage is showing the cumulative incidence, and 95% confidence intervals are based on Kaplan–Meier survival analysis. (—— first infection; − - - - second infection; — • • — third infection; — - - — fourth infection).

**Figure 3:**
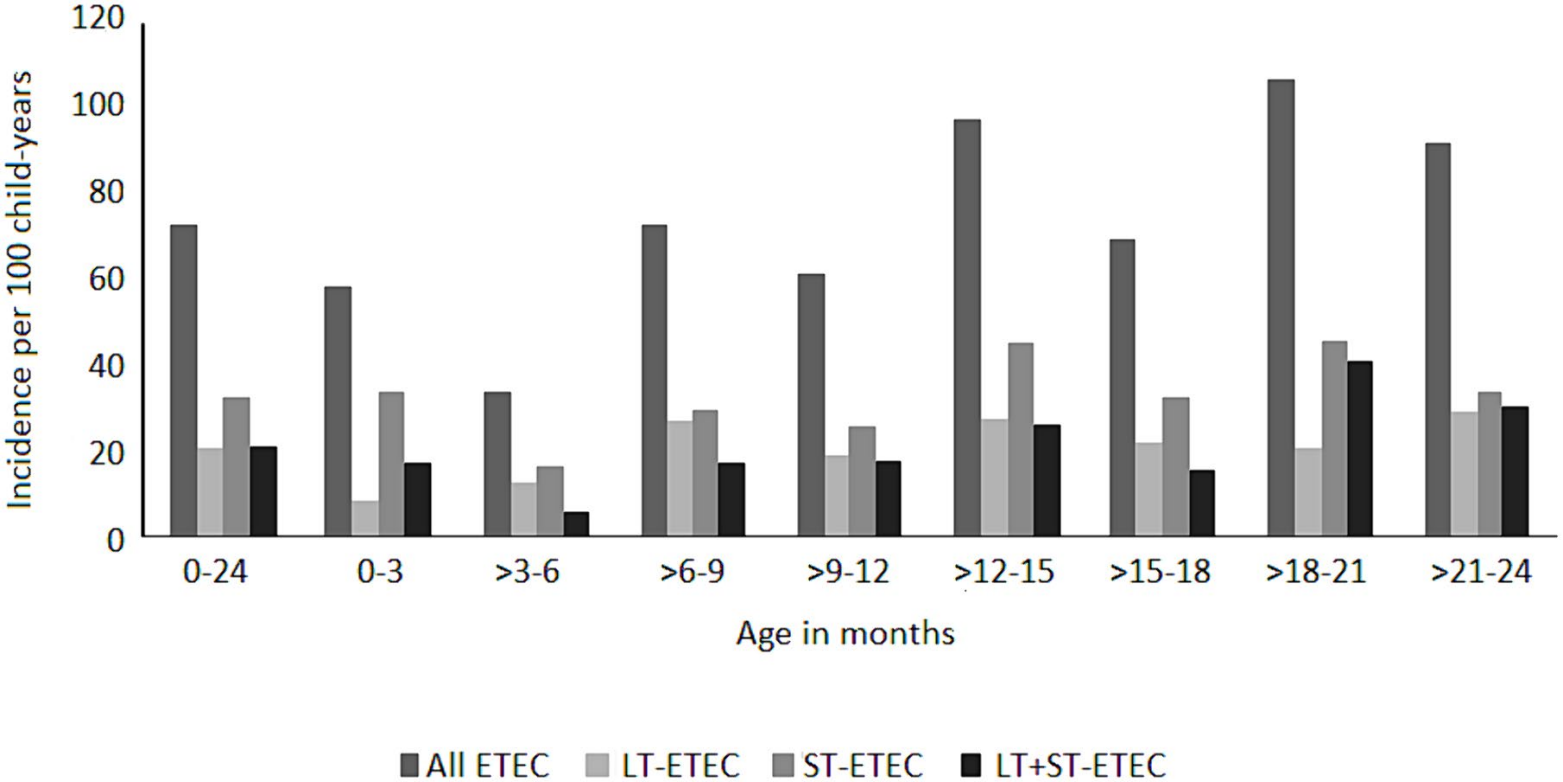
Incidence of ETEC diarrhea episodes by age stratum. Incidence of ETEC diarrhea per 100 child-years by the type of toxins at different age groups at three months intervals. “0–24” shows the two years follow up period.

ETEC diarrhea incidence started early after birth and markedly increased with age, while ETEC negative diarrhea incidence decreased after 200 days of life (Figure 1). The overall incidence rate per 100 child years (IR) of ETEC positive diarrhea was 72.9 episodes. By the 1^st^ year of life, 33.8% and by the 2^nd^ year of life, 68% of the children suffered from one or more (up to 7) ETEC diarrhea episodes (Figure 2A). The frequency of two or more ST-ETEC diarrhea episodes (46.4%) per child was higher than LT-ETEC (29.8%) and LT + ST-ETEC (23.8%) (Figures 2B–D; Table 3). The ETEC diarrhea IR was significantly higher in the second year of life than in the first year (91.2 vs. 56.1; *p* = 0.023, 95% CI 6.7–62.9%) (Figure 3). The highest IR 106.4 was at 18–21 months of age. The overall IR of ST-ETEC (32.1) was highest, followed by LT + ST-ETEC (20.6) and LT-ETEC (20.2) (Figure 3). The IR of STp-ETEC was 9.3.

**Table 3:** Frequency of repeated ETEC diarrhea episodes by the toxin types in children in the 2 years of follow up.

Number of ETEC infections per child	Number of children	Number of ETEC infections	LT-ETEC infections (*n*) *n*/number of ETEC infections (%)		ST-ETEC infections (*n*) *n*/number of ETEC infections (%)		LT + ST-ETEC infections (*n*) *n*/number of ETEC infections (%)	
2	66	132	39	29.5%	64	48.5%	29	22.0%
3	60	180	51	28.3%	86	47.8%	43	23.9%
4	31	124	38	30.6%	53	42.7%	33	26.6%
5	22	110	37	33.6%	48	43.6%	25	22.7%
6	10	60	18	30.0%	33	55.0%	9	15.0%
7	4	28	6	21.4%	10	35.7%	12	42.9%
Total	193	634	189	29.8%	294	46.4%	151	23.8%

Children who had two or more ETEC diarrhea and completed 2 years of follow up *n* = 193.

**Figure 4:**
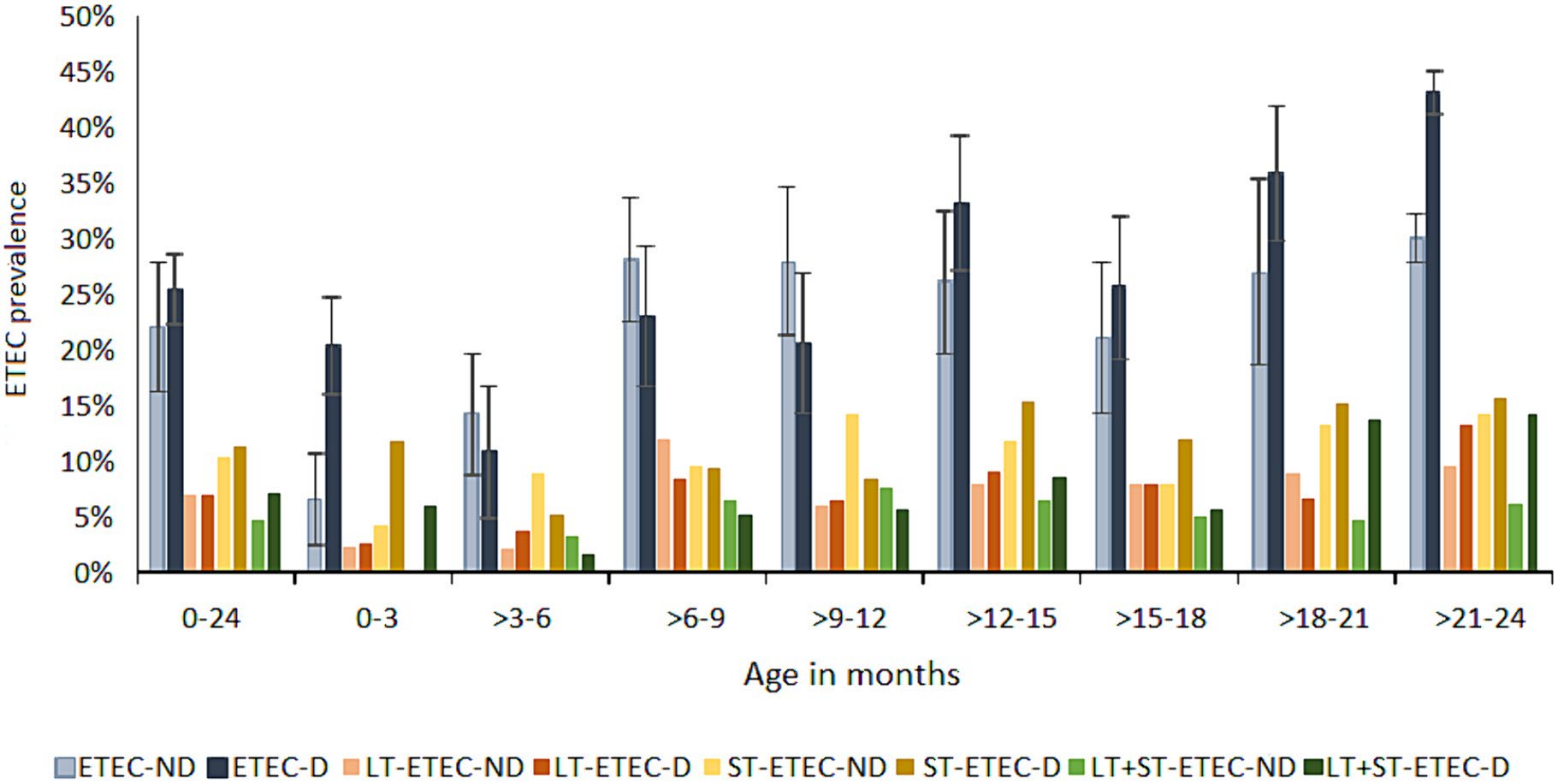
Prevalence of ETEC in diarrhea and asymptomatic routine surveillance samples. ETEC prevalence in diarrhea and non-diarrhea samples by toxin types at three months of age intervals. 0–24: the prevalence of ETEC in the total follow up period of 24 months in each child. Error bars showing 95% confidence intervals. ND: no diarrhea; D: diarrhea.

The ETEC attributable diarrheal burden (PAF) over 2 years was 5.2% (2–8.3) (Figure 4, see Supplementary Table S3), which widely varied by age. Notably, the ETEC PAF was 10% (8.9–11.1) in the neonates (0–3 months), and most of the infections (58%) were attributed to ST-ETEC [PAF 7.9% (1.9–13.5)] and LT + ST-ETEC (29%) of which all the episodes were associated with diarrhea. Of the ST-ETEC diarrhea, 77% was contributed by the STh and STh + STp-ETEC. ETEC was increasingly associated with asymptomatic infections in the subsequent age strata in the first year of life. At 3–6 months, the prevalence of ETEC infections was the lowest of all age stratum and was primarily contributed by ST-ETEC (48%) and LT-ETEC (36%). The LT-ETEC PAF [2.8% (1.9–3.7)] was significant only in the 3–6 months of age. The prevalence of the ETEC toxin types was equally distributed in 6–12 months of age. The ETEC PAF significantly increased at the beginning of the second year of life, with the highest being 17% (4.6–27.7) at the age of 21–24 months. In fact, at 18–24 months of age, 40% of the total diarrhea episodes was positive for ETEC, mostly attributed to LT + ST-ETEC [PAF 11.6% (4.8–12.3) at 18–21 months and 11.9% (1.1–11.9), at 21–24 months]. The overall STp-ETEC PAF was 1.4% (0.7–2.2) and was significant at the 6–9 months [2.7% (1.4–4.0)] and 12–15 months [4.1% (2.0–6.2)] age strata.

Among the first diarrhea episodes in children, 52 (17%) were positive for ETEC, with the youngest child being five days old. The mean age of the first ETEC diarrhea episode was 9.4 months (min 0.2, max 23.9). Of the 122 neonates who had their first diarrhea episode within three months of age, 21 (17.2%) were positive for ETEC, of which 13 (61.9%) were ST-ETEC (Table 4).

**Table 4:** First ETEC diarrhea episodes by age strata.

Age stratum (months)	Number of children with first diarrhea episode positive for ETEC				
	First diarrhea episodes (total number)	ETEC diarrhea *n* (%)	LT-ETEC *n* (%)	ST-ETEC *n* (%)	LT + ST-ETEC *n* (%)
0–3	122	21 (17.2)	2 (1.6)	13 (10.7)	6 (4.9)
3–6	81	8 (9.9)	3 (3.7)	3 (3.7)	2 (2.5)
6–9	51	11 (21.6)	5 (9.8)	4 (7.8)	2 (3.9)
9–12	17	2 (11.8)	1 (5.9)	1 (5.9)	0
12–15	13	4 (30.8)	0	3 (23.1)	1 (7.7)
15–18	7	2 (28.6)	2 (28.6)	0	0
18–21	10	3 (30)	1 (10)	0	2 (20)
21–24	4	1 (25)	0	1 (25)	0
Total	305	52 (17)	14 (4.6)	25 (8.2)	13 (4.3)

The most frequent co-pathogens among the ETEC diarrhea cases were *Campylobacter* spp. (25%), sapovirus (22%) and *Shigella* spp. (13.2%) and among the ETEC asymptomatic cases, *Campylobacter* spp. (13.7%) and norovirus GII (8.2%) (see Supplementary Table S4).

### Duration of shedding of ETEC following diarrhea and asymptomatic ETEC cases

**Figure 5:**
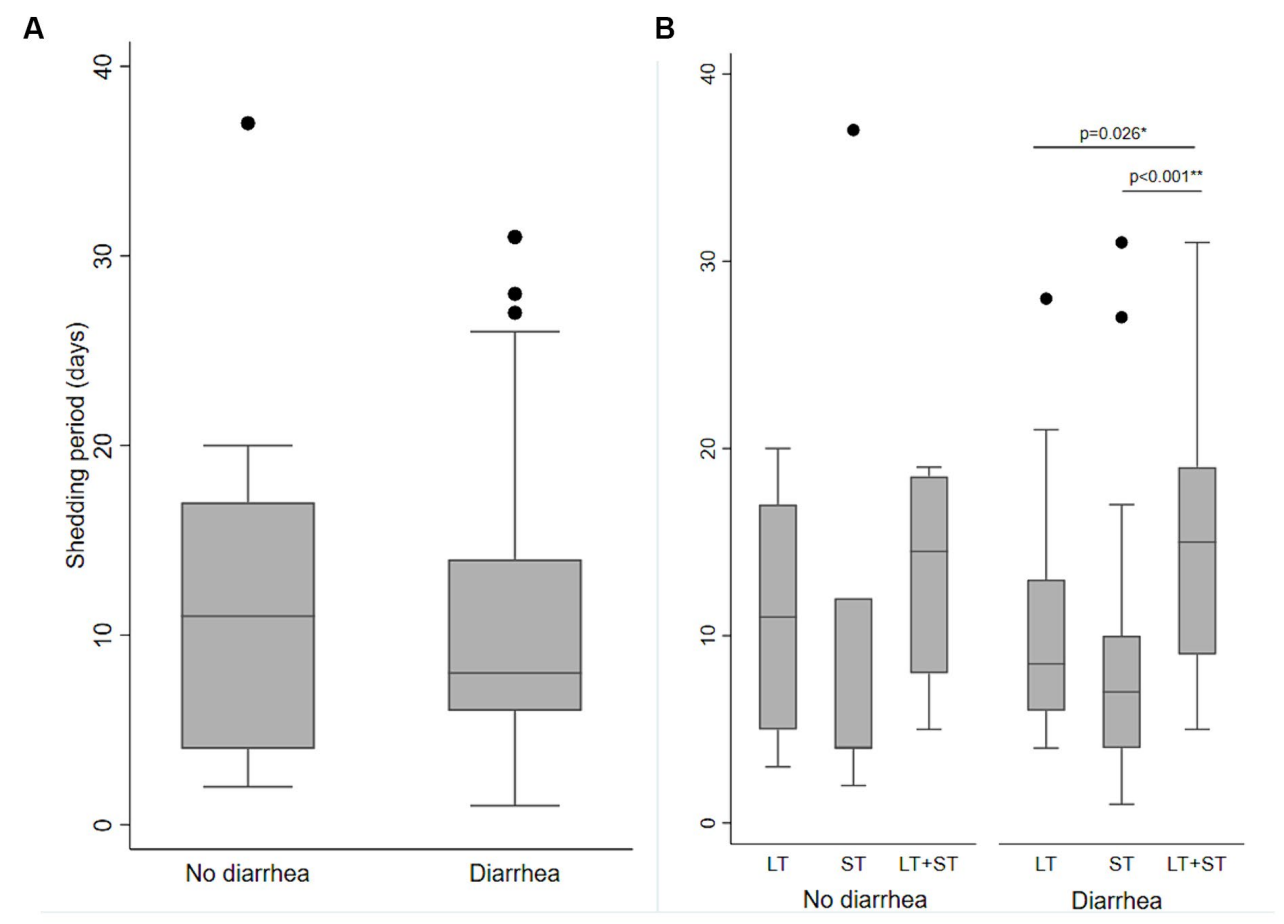
Shedding of ETEC following ETEC diarrhea and asymptomatic infections. **(A)** Length of shedding of ETEC following ETEC infections in the diarrhea and no diarrhea episodes. **(B)** Length of shedding of ETEC by the ETEC toxin types following ETEC infections in diarrhea and no diarrhea episodes. Significant differences are shown by Kruskal Wallis test and then Dunn test for pairwise comparison, **(A)** no diarrhea vs. diarrhea; **(B)** LT-ETEC vs. ST-ETEC and LT-ETEC vs. LT + ST-ETEC.

Ninety ETEC episodes [diarrhea (*n* = 70) and asymptomatic (*n* = 20)] were randomly selected, and weekly stool samples (total *n* = 189) were tested for ETEC following each of those episodes until two consecutive stools were negative for ETEC with the same toxin types. The mean shedding duration of ETEC was 10.6 days (SD 7.4, range 1 to 37 days), which was similar between diarrhea and asymptomatic cases (median 10.7 and 10.6 days; SD 8.5 and 7.1; *p* = 0.601). Among the diarrhea episodes, the shedding was significantly longer for LT + ST-ETEC episodes (mean 15.4 days, SD 7.3) compared to LT-ETEC (mean 10.1, SD 5.9, *p* = 0.026) and ST-ETEC (mean 8.3 days; SD 7.2; *p* < 0.001) (Figure 5).

### ETEC colonization factors and serogroups

**Figure 6:**
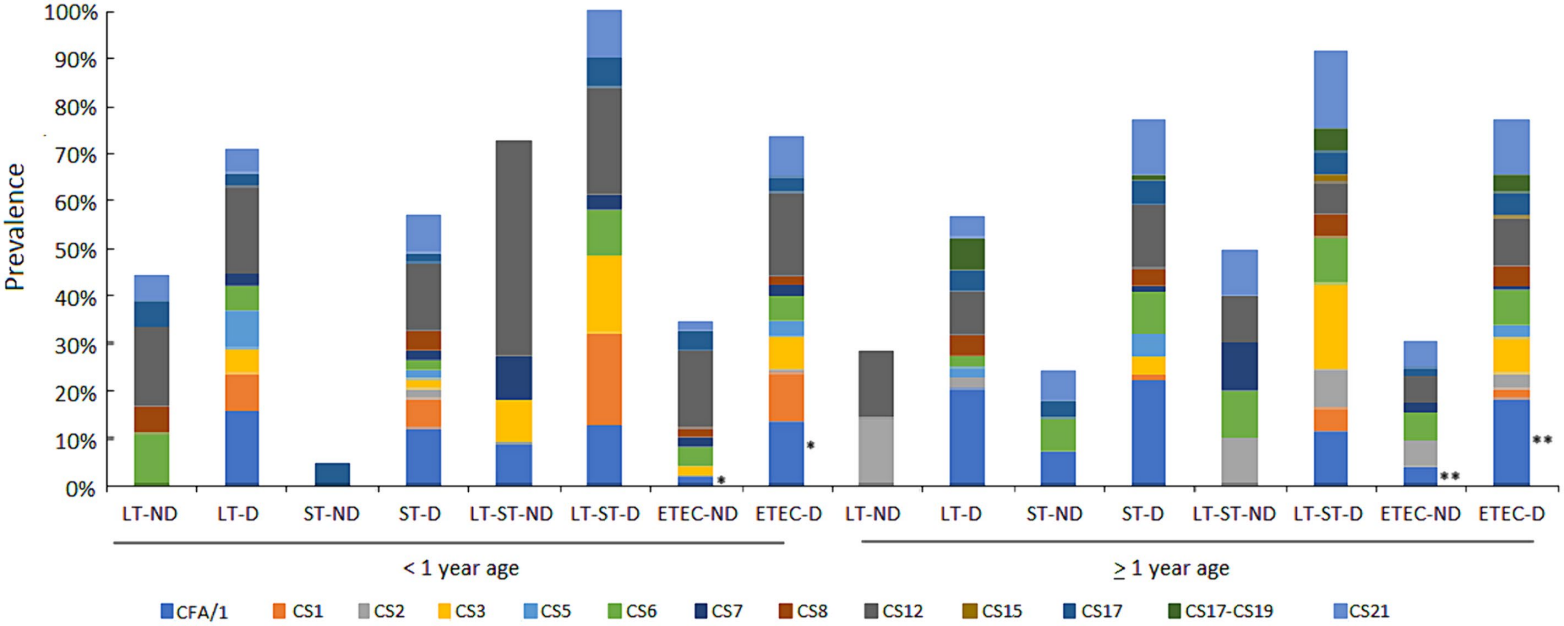
Proportion of ETEC CFs by toxin types in diarrhea and asymptomatic cases in the first and second year of life. The prevalence of CFs and associations with the ETEC toxin types by ETEC diarrhea and ETEC no diarrhea episodes among the children less than 1 year and above 1 year of age. Comparisons of CFs among diarrhea and asymptomatic cases was performed using GEE population averaged model to adjust for repeated measures. *A marginal difference was found in children less than 1 year *p* = 0.058 and ** a significant difference was found in children above 1 year of age *p* = 0.023 for CFA/I.

CFs were detectable in 212 (69.7%) of the 304 ETEC diarrhea episodes tested. Overall, CFA/I (16.4%) was the most commonly occurring *CF*, followed by CS12 (13.2%), CS21 (10.5%), CS3 (7.2%) and CS6 (6.6%). Among 101 asymptomatic ETEC cases tested, CFs could not be detected in 30 (29.7%). The most commonly occurring CFs were CS12 (10.9%) followed by CS6 (5%) and CS21 (4%). The prevalence of CFs and their association with toxins by age and clinical outcome (diarrhea or non-diarrhea) are shown in Figure 6.

The ETEC infection was significantly associated with diarrhea when CFs were detected (OR:3.37, *p* < 0.001). Only CFA/I ± CS21 was significantly associated with ETEC diarrhea episodes [OR = 3.97 (1.76–9.12, *p* = 0.001)] and was more prevalent in children >12 months old [OR = 1.96 (1.14–3.35, *p* = 0.029)], all adjusted for intra-child correlation.

Among the 44 ETEC isolates tested, 20 different O serogroups were detected, of which O148 was detected most frequently (18.2%), followed by O164 and O158 (9.1% each), and O128 and O125 (6.8% each).

### Repeat infections with ETEC

Although 193 children had serial ETEC infections, only 11 (5.7%) had repeat infections with the same combination of ETEC toxin(s) and CFs. We analyzed if a primary ETEC infection prevented reinfection with ETEC of a homologous toxin or *CF* type within three months of the first infection. Because of the small number, although no significant homologous protection was noted for any toxin and CFs, a longer intervals of CFA/I ETEC infections, regardless of toxins was noted when previous ETEC infection was with CFA/I. The median time between the repeat infections with CFA/I was 269 days, while that for CS21, CS12, CS3, and CS17 were 36, 29.5, 14 and 6 days. There was no repeat infection with CS6. There were more repeat infections of ST-ETEC, regardless of CFs in the same child [49.35%; (39.0–59.7)] compared to LT-ETEC [24.68%; (16.4–35.4)] or LT + ST-ETEC infections [25.97%; (17.6–36.5)].

### The impact of ETEC on growth

Out of 332 children whose birth weight could be collected, 10 (3%) were born underweight (< 2.5 kg; range 1.7 to 2.49) and of 314 children who completed 3 m of follow up, 15 (4.7%) were stunted (z-score height for age < −2.0) at 3 months of age.

We evaluated the impact of ETEC diarrhea on the immediate and long-term growth of the children. Using a velocity model, for an average ETEC diarrhea episode of 5.3 days in the previous measurement interval (approximately one month), there was a reduction of 0.060 (0.007 to 0.114; *p* = 0.027) in the weight for length Z-score (WLZ) and 0.061 (0.015 to 0.108; *p* = 0.009) in the weight for age z-score (WAZ) in the following 30 day interval, compared to no ETEC infection (see Supplementary material for details). The total number of ETEC diarrhea episodes over two years of follow up was not significantly associated with WAZ (coefficient = 0.032 (−0.074 to 0.139, *p* = 0.547) or length for age z-score (LAZ) (coefficient = 0.045 (−0.053 to 0.144, *p* = 0.366) measured at the end of the follow-up, adjusted by sex, age at the end of follow up and birth weight (Supplementary Tables S5–S8).

## Discussion

In this peri-urban shantytown, ETEC diarrhea began as early as five days after birth, and the burden was substantial, infecting about 70% of the children by the time they were two years old and having up to seven ETEC diarrhea episodes, impacting the growth of the children. Most ETEC diarrhea was moderate to severe, and a considerable proportion was associated with fever and vomiting.

The incidence rates and diarrheal episodes attributed to ETEC among the neonates were significantly high, and notably, most infections were attributed to ST-ETEC. ST is poorly immunogenic, and therefore neonates may have suboptimal protection against ST-ETEC infections through maternal immunity. The immune responses to the majority of the CFs from natural ETEC infections are generally low and may need repeated exposures to achieve optimum immunity (21, 34). To protect the vulnerable neonatal period, an ETEC vaccine that includes ST antigen and overexpressed *CF* antigens to achieve increased immunity, as in the ETVAX ETEC vaccine (11, 12), could be given at birth or as a maternal vaccine. The later immunization route could avoid the challenge of low immunity in infants from oral vaccines.

The decrease in total ETEC infections at 3–6 months and increase in asymptomatic ETEC infections in the first year of life, with equal distributions of the toxin types, likely reflect the protection from transferred maternal antibodies through breastfeeding as well as immunity from past infections in children. In the second year of life, following weaning to complementary foods, ETEC infections steadily increased, accounting for about half of the total diarrhea cases, mostly attributable to LT + ST-ETEC, and were more strongly associated with diarrhea than in the first year.

Overall, ST-ETEC was the most frequent in this cohort, followed by LT + ST-ETEC, which had the longest shedding. LT-ETEC, although highly prevalent in all age groups, was associated on average equally with diarrhea and asymptomatic infections, which could be a result of the higher immunogenicity of LT (35) and the protection from prior LT-ETEC infections, which resulted in fewer repeat infections with LT-ETEC in the same child. As reported before, STp-ETEC was detected at higher frequencies than in Asian and some African countries (36, 37). Notably, STp-ETEC contributed 15% of the ETEC diarrhea episodes and was significantly attributed to diarrhea in at least two age strata, which signifies the importance of this ETEC pathotype in causing diarrhea. Consistent with prior studies from Latin American countries (38, 39), minor CF, CS21, was frequently detected in the ETEC diarrhea cases. CS21 pilus had shown to contribute to adhesion to intestinal cells and to pathogenesis under *in vivo* conditions (40). Another minor CF, CS12, was detected in both diarrheal and asymptomatic cases. Notably, CS21 and CS12 are not included in the advanced vaccine candidates (10, 11). Although repeated ETEC infections were frequent in children, repeat infections by the same toxin (other than ST) and CF types of ETEC were rare.

Our study population appears to have had less acute malnutrition at birth than those in South Asia (37, 41). However, ETEC diarrhea had a significant negative impact on the short-term growth of the children.

This study has several strengths. This is the first birth cohort study of ETEC in Lima, which is geographically distinct from the Peruvian Amazon, one of the sites in the MAL-ED study (42). We conducted frequent home visits to ensure all episodes of diarrhea were captured, and we tested samples frequently for asymptomatic infections. Study limitations should be acknowledged. We included stringent requirements to define the end of infection episodes and their association with diarrhea; our incidence estimates are therefore conservative. We tested 14 CFs which although includes all the major CFs except CS14 (43), we may have missed some minor CFs circulating in this area. Limited testing for other enteric pathogens in this study means that some study children with ETEC likely had undetected co-pathogens contributing to the synergistic effect on pathogenicity, although the impact of individual co-pathogen in a diarrhea episode is difficult to determine and depends on multiple factors. In addition, as reported before, the incidence of mixed infections seems to increase with age and fewer co-pathogens were seen in infants than in older children and adults with ETEC diarrhea (1).

## Conclusion

This study highlights the significance of ETEC for Peruvian children in Lima and underscores the importance of the development and implementation of ETEC vaccine. The high neonatal ETEC burden noted in our study suggests that an ETEC vaccine should be given at birth or as a maternal vaccine. To achieve considerable protection from ETEC in this area, the current vaccine candidates need to include CS21 and CS12, along with ST. The serial ETEC infections by multiple genotypes in a child suggest genotype-specific immunity from each infection, which should be considered when developing vaccines. The data from this study will strengthen modeled disease burden estimates and facilitate the design of improved vaccines to prevent ETEC diarrhea and infections.

## Data availability statement

The original contributions presented in the study are included in the article/Supplementary material, further inquiries can be directed to the corresponding author.

## Ethics statement

The studies involving humans were approved by Institutional review boards of Asociación Benéfica PRISMA, Universidad Peruana Cayetano Heredia, and Johns Hopkins University. The studies were conducted in accordance with the local legislation and institutional requirements. Written informed consent for participation in this study was provided by the participants’ legal guardians/next of kin.

## Author contributions

MP: Writing – original draft, Writing – review & editing. SN: Writing – review & editing. LC: Writing – review & editing. AT: Writing – review & editing. MV: Writing – review & editing. LA: Writing – review & editing. MO: Writing – review & editing. LM: Writing – review & editing. MS: Writing – review & editing. RG: Writing – review & editing. SC: Writing – original draft, Writing – review & editing.
