# Supplementary material for: Epidemiology of enterotoxigenic Escherichia coli and impact on the growth of children in the first two years of life in Lima, Peru
Source: Front Public Health. 2024 Mar 22;12:1332319. doi: 10.3389/fpubh.2024.1332319 (PMC10995271; doi:10.3389/fpubh.2024.1332319)
Supplement: Supplementary file 1 [file Data_Sheet_1.docx]

**Supplement data:**

**Definitions.** Diarrheal episodes were identified based on the primary caregiver's report through daily home visits by the fieldworkers. A diarrhea episode was defined as >3 liquid stools in 24 hours and were separated by 2 consecutive diarrhea-free days. Diarrheal episodes of <7 days duration was classified as acute, and >7 days as persistent. Data on the components for diarrhea severity based on CODA index were collected. Severe diarrhea with an index of >7, moderate diarrhea between 1 and 6 and mild diarrhea if the index was 0. Fever (yes/no) was recorded as per the caregiver’s observation. LT-ETEC is represented by ETEC with only LT; ST-ETEC by ETEC with STh or STp or both; and LT+ST-ETEC is by ETEC with LT and either of STh or STp or both. All data were analyzed using STATA.

**Supplementary Table 1. The incidence rates of ETEC diarrhea comparing the cycle thresholds (Ct) cutoffs of 35 and 40.**

|  | **Cutoff of Ct=35** | | | | **Cutoff of Ct=40** | | | |
| --- | --- | --- | --- | --- | --- | --- | --- | --- |
| Age category (months) | All ETEC | LT-ETEC | ST-ETEC | LT+ST-ETEC | All ETEC | LT-ETEC | ST-ETEC | LT+ST-ETEC |
| 0-3 | 55.15 | 7.66 | 32.17 | 15.32 | 58.21 | 7.66 | 33.70 | 16.85 |
| 3-6 | 28.25 | 10.77 | 12.11 | 5.38 | 33.63 | 12.11 | 16.14 | 5.38 |
| 6-9 | 65.71 | 25.17 | 25.17 | 15.38 | 72.7 | 26.57 | 29.36 | 16.78 |
| 9-12 | 56.75 | 24.74 | 21.89 | 10.19 | 61.12 | 18.92 | 24.74 | 17.46 |
| 12-15 | 91.68 | 27.05 | 43.58 | 21.04 | 97.69 | 27.05 | 45.09 | 25.55 |
| 15-18 | 53.59 | 18.37 | 22.97 | 12.25 | 68.9 | 21.44 | 32.15 | 15.31 |
| 18-21 | 98.60 | 17.22 | 51.65 | 29.74 | 106.43 | 20.35 | 45.39 | 40.7 |
| 21-24 | 79.15 | 18.97 | 33.24 | 26.91 | 91.82 | 28.5 | 33.24 | 30.08 |
| Total | 65.33 | 18.75 | 29.88 | 16.70 | 72.95 | 20.23 | 32.11 | 20.60 |

**Supplementary Figure 1.** **Daily incidence proportion of ETEC positive and ETEC negative diarrhea episodes**

**A**

**
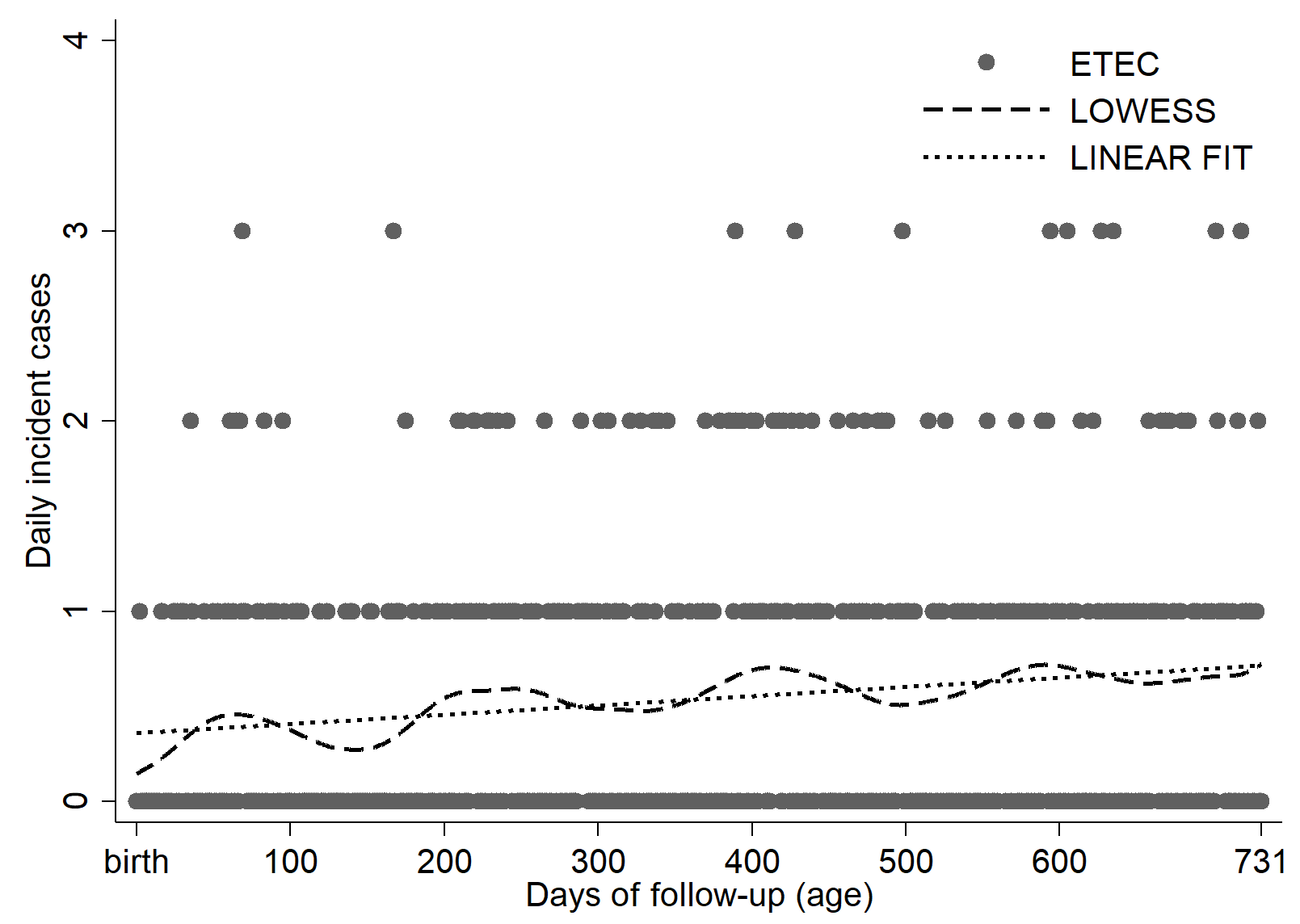
**

**B**

**
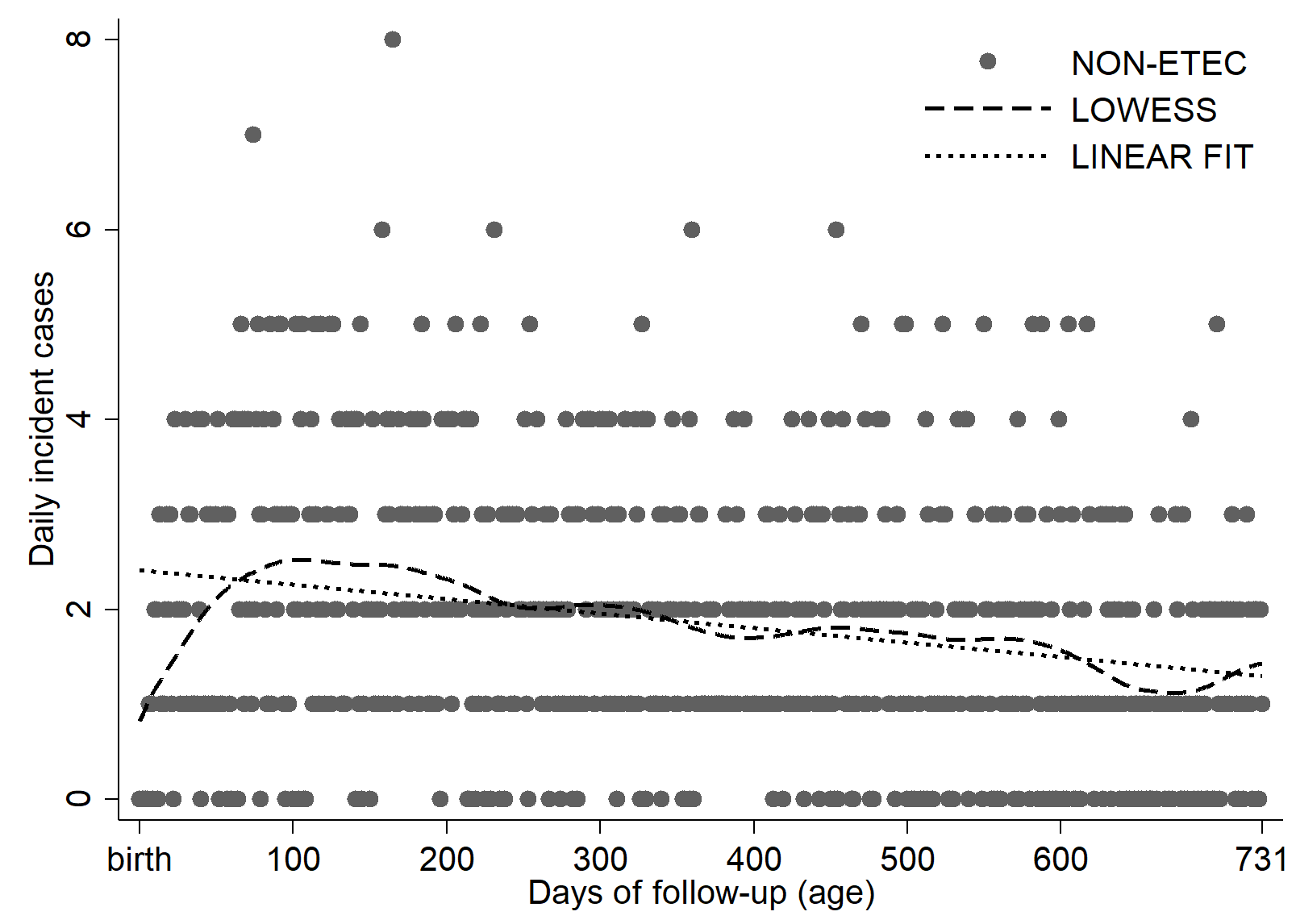
**

Legend: Figure 1A. ETEC diarrhea daily incidence proportion over the study duration. Graph indicates incident cases by days of follow-up, the LOWESS smoothed curve (bandwidth=0.2) and the linear trend. Figure 1B. Non-ETEC diarrhea daily incident cases over the study duration. Graph indicates the incident cases by days of follow-up, the LOWESS smoothed curve (bandwidth=0.2) and the linear trend.

**Supplementary Table 2. ETEC incidence per person-year by secretor status**

|  | N | mean | sd | p25 | p50 | p75 | min | max |
| --- | --- | --- | --- | --- | --- | --- | --- | --- |
| Secretor | 329 | 1.6 | 1.6 | 0.5 | 1.5 | 2.1 | 0.0 | 19.2 |
| Non secretor | 5 | 1.9 | 1.7 | 0.0 | 3.1 | 3.1 | 0.0 | 3.3 |
| Total | 334 | 1.6 | 1.6 | 0.5 | 1.5 | 2.1 | 0.0 | 19.2 |

**Association of blood group and ETEC diarrhea:** Of the 277 children with blood group detected, 12.6% (n=35) were blood type A, 7.2% (n=20) were type B, and 80% (n=221) were type O (Supplement Figure 1). The majority, 98.5% (329 of 334) of the children, were secretors (Supplement Table 4). No significant association was noted of any specific blood group or secretor status with the number of ETEC diarrhea or severity of diarrhea.

**Supplementary Figure 1. ETEC diarrhea incidences by blood groups**


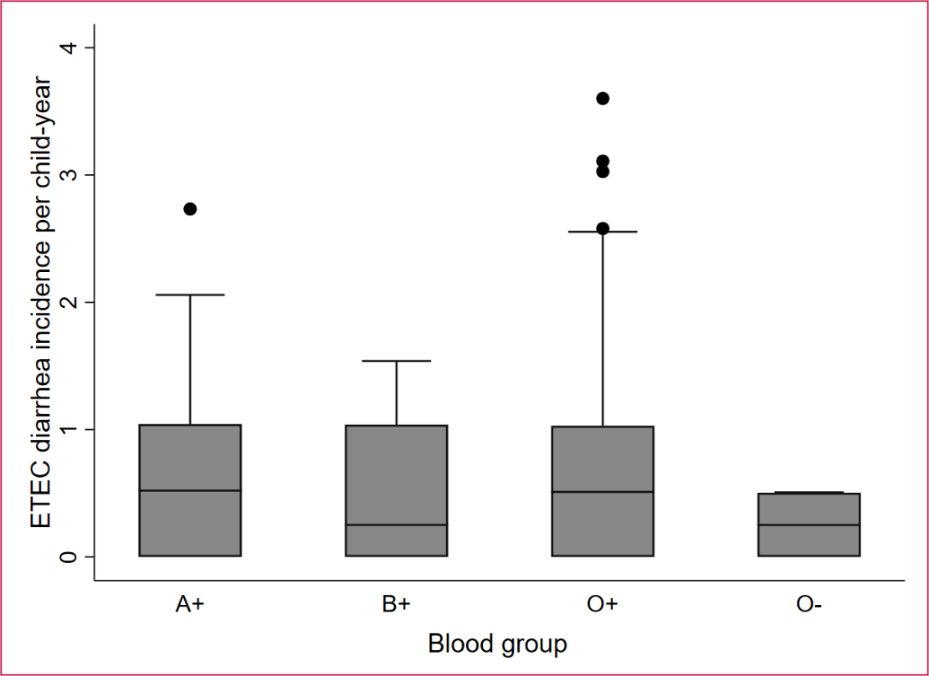


Legend: Box plot of ETEC diarrhea incidence per child year by the blood group of the children.

**Supplementary Table 3. ETEC diarrhea population attributable fractions (PAF)**

|  | OR | *p value* | Population attributable fraction [95% CI] |
| --- | --- | --- | --- |
| Overall in 2 years | | | |
| ETEC | 1.250 | 0.004 | 5.19% (2.00% - 8.30%) |
| LT+ST-ETEC | 1.560 | 0.002 | 2.60% (1.30% - 3.90%) |
| STp-ETEC | 1.769 | 0.007 | 1.41% (0.65% - 2.17%) |
| 0-3Months | | | |
| ETEC | 1.953 | <0.001 | 9.97% (8.83% - 11.10%) |
| ST-ETEC | 2.976 | 0.006 | 7.85% (1.89% - 13.46%) |
| 3-6Months | | | |
| LT-ETEC | 3.500 | 0.002 | 2.81% (1.90% - 3.71% |
| 6-9Months | | | |
| STp-ETEC | 4.089 | 0.057 | 2.69% (1.42% - 3.94%) |
| 12-24Months | | | |
| ETEC | 1.503 | 0.000 | 11.40% (6.38% - 16.16%) |
| LT+ST-ETEC | 2.122 | <0.001 | 8.49% (3.64% - 7.31%) |
| 12-15Months | | | |
| STp-ETEC | 3.052 | 0.034 | 4.14% (2.03% - 6.20%) |
| 15-18Months | | | |
| STh-ETEC | 2.420 | 0.046 | 5.36% (2.02% - 8.59%) |
| 18-21Months | | | |
| ETEC | 1.461 | 0.082 | 11.35% (0.18% - 21.28%) |
| LT+ST-ETEC | 2.671 | 0.008 | 11.61% (4.77% - 12.28%) |
| 21-24Months | | | |
| All ETEC | 1.644 | 0.026 | 16.96% (4.56% - 27.73%) |
| LT+ST-ETEC | 1.882 | 0.083 | 11.93% (1.10% - 11.88%) |

The two-sided 95% CIs for the PAFs was estimated using the PUNAF Stata module. (Newson, Roger B. Attributable and unattributable risks and fractions and other scenario comparisons. The Stata Journal. Volume 13 Number 4: pp. 672-698). Only the significant PAF data are added in the table.

**Supplementary Table 4. Co-pathogens among ETEC cases**

| Total | norovirus GI | | norovirus GII | | sapovirus | | Shigella | | Campylobacter | |
| --- | --- | --- | --- | --- | --- | --- | --- | --- | --- | --- |
|  | Stool tested N | N (%) | Stool tested N | N (%) | Stool tested N | N (%) | Stool tested N | N (%) | Stool tested N | N (%) |
| Total ETEC cases | 147 | 5 (3.4%) | 148 | 13 (8.8%) | 148 | 17 (11.5%) | 78 | 8 (10.3%) | 129 | 14 (10.85%) |
| ETEC asymptomatic cases | 98 | 3 (3.1%) | 98 | 8 (8.2%) | 98 | 6 (6.1%) | 25 | 1 (4%) | 73 | 10 (16.4%) |
| ETEC diarrhea cases | 50 | 2 (4%) | 50 | 5 (10%) | 50 | 11 (22%) | 53 | 7 (13.2%) | 56 | 14 (25%) |

**Analysis of ETEC diarrhea and growth of children**

Children with low birth weight or stunted at 3 months of age had a higher number and longer duration of ETEC diarrhea compared to healthy children, although the associations were not significant. Analysis was done separately for low birthweight: p=0.473 for number of episodes and p=0.613 for total days of ETEC diarrhea; for stunting at 3 months: p=0.174 for number of episodes and p=0.218 for total number of days of ETEC diarrhea.

We evaluated the association of the change in anthropometry in the interval between two consecutive measurements with the proportion of ETEC diarrhea days during the measurement interval immediately preceding it (intervals of approximately one month). To do this, we fitted velocity models separately for WAZ, WLZ and LAZ using generalized estimation equations (GEE) with robust variance (xtgee, Stata 14) to account for the within-child correlation due to repeated measurements. The outcome variable was the average daily change in anthropometric measurements, defined as the change between two consecutive measurements (at time t_0_ and time t_1_) divided by the number of days in that interval. The main predictor was the average daily ETEC diarrhea duration in the immediately preceding interval (between time t_-1_ and time t_0_), defined as the total number of days of ETEC diarrhea divided by the number of days in the interval. While the association was significant for WAZ and WLZ (Supplement Tables 5 and 6), it was not for LAZ.

**Supplement Table 5. Association of change in WAZ and the proportion of ETEC diarrhea days during the previous measurement period.**

| **WAZ change velocity*** | **Coef.** | **Std.Err** | **z** | **P>\|z\|** | **[95% Conf. Interval]** | |
| --- | --- | --- | --- | --- | --- | --- |
| Birth WAZ | 0.003 | 0.001 | 4.44 | <0.001 | 0.002 | 0.004 |
| WAZ in previous measurement** | -0.011 | 0.001 | -10.01 | <0.001 | -0.013 | -0.009 |
| Age (months) | -0.0004 | 4.33E-5 | -8.82 | <0.001 | -0.0005 | -0.0003 |
| ETEC proportion previous period*** | -0.012 | 0.004 | -2.60 | <0.009 | -0.020 | -0.003 |
| Constant | 0.009 | 0.001 | 8.13 | <0.001 | 0.007 | 0.011 |

* Average daily change in WAZ in current anthropometric measurement period

**WAZ measured in previous measurement period

***Proportion of ETEC diarrhea days during previous measurement period

**Supplement Table 6. Association of change in WLZ and the proportion of ETEC diarrhea days during the previous measurement period**

| **WLZ change velocity*** | **Coef.** | **Std.Err** | **z** | **P>\|z\|** | **[95%**  **Conf. Interval]** | |
| --- | --- | --- | --- | --- | --- | --- |
| Birth WLZ | 0.002 | 0.001 | 2.48 | <0.013 | 0.000 | 0.003 |
| WLZ previous measurement** | -0.014 | 0.001 | -22.45 | <0.001 | -0.015 | -0.013 |
| Age (months) | -0.0003 | 4.35E-05 | -7.82 | <0.001 | -0.0004 | -0.0003 |
| ETEC proportion previous period*** | -0.011 | 0.005 | -2.22 | <0.027 | -0.022 | -0.001 |
| constant | 0.018 | 0.001 | 16.53 | <0.001 | 0.016 | 0.020 |

* Average daily change in WLZ in current anthropometric measurement period

** WLZ measured in previous measurement period

***Proportion of ETEC diarrhea days during previous measurement period

The association between the number of ETEC diarrhea episodes during the follow up and the last anthropometric measurement (WAZ, WLZ or LAZ) at the end of follow up was assessed by fitting linear regression models (separately for WAZ, WLZ and LAZ) with the last anthropometric measurement as the outcome (Supplement Tables 7 and 8). These models showed a significant negative association between the number of ETEC episodes during the follow-up with WAZ (prob F =0.0000, n=327) and LAZ (prob F=0.0000, n=326), among boys (p=0.049 for WAZ and p=0.022 for LAZ). Association was not significant among girls, for WAZ (p=0.365) or LAZ (p=0.080). Models exploring these associations include birth weight, age at the end of follow-up, sex, the number of ETEC episodes and the interaction between sex and the number of ETEC episodes. The model for WLZ did not show a significant association.

**Supplement Table 7.** **Association of final weight measurement (WAZ) and the number of ETEC diarrhea episodes. Linear regression model adj R2=0.092, prob F =<0.0001, n=327.**

| **Final WAZ** | **Coef.** | **Std. Err** | **t** | **P>\|t\|** | **95%**  **Conf. Interval** | |
| --- | --- | --- | --- | --- | --- | --- |
| Birth weight | 0.523 | 0.110 | 4.77 | <0.001 | 0.307 | 0.739 |
| Number of ETEC diarrhea (female) | 0.044 | 0.049 | 0.91 | <0.365 | -0.052 | 0.140 |
| Sex | 0.112 | 0.130 | 0.86 | <0.389 | -0.143 | 0.367 |
| Number of ETEC diarrhea interaction with sex (male) | -0.142 | 0.072 | -1.97 | <0.049 | -0.283 | -0.001 |
| Final age (months) | 0.000 | 0.000 | -1.73 | <0.085 | -0.001 | 0.000 |
| Constant | -1.281 | 0.405 | -3.17 | <0.002 | -2.077 | -0.485 |

**Supplement Table 8.** **Association of final length measurement (LAZ) and the number of ETEC diarrhea episodes. Linear regression model adj R2=0.197, prob F=0.0000, n=326.**

| **Final LAZ** | **Coef.** | **Std. Err** | **t** | **P>\|t\|** | **95%**  **Conf. Interval** | |
| --- | --- | --- | --- | --- | --- | --- |
| Birth Length | 0.197 | 0.026 | 7.57 | <0.001 | 0.146 | 0.248 |
| Number of ETEC diarrhea (female) | 0.080 | 0.046 | 1.76 | <0.080 | -0.010 | 0.170 |
| Sex | -0.074 | 0.121 | -0.61 | <0.545 | -0.312 | 0.165 |
| Number of ETEC diarrhea interaction with sex (male) | -0.155 | 0.067 | -2.3 | <0.022 | -0.288 | -0.023 |
| Final age (months) | -0.029 | 0.007 | -4.19 | <0.001 | -0.043 | -0.016 |
| Constant | -9.855 | 1.304 | -7.56 | <0.000 | -12.420 | -7.290 |
